# Supplementary material for: Single-nucleus mRNA-sequencing reveals dynamics of lipogenic and thermogenic adipocyte populations in murine brown adipose tissue in response to cold exposure
Source: Mol Metab. 2025 Sep 11;101:102252. doi: 10.1016/j.molmet.2025.102252 (PMC12506565; doi:10.1016/j.molmet.2025.102252)
Supplement: Multimedia component 4 [file mmc4.pdf]

S1A

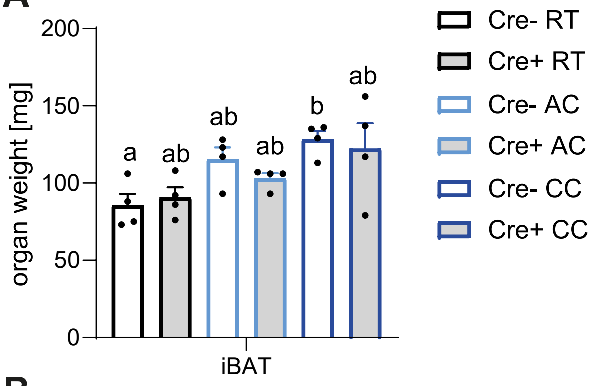

S1B

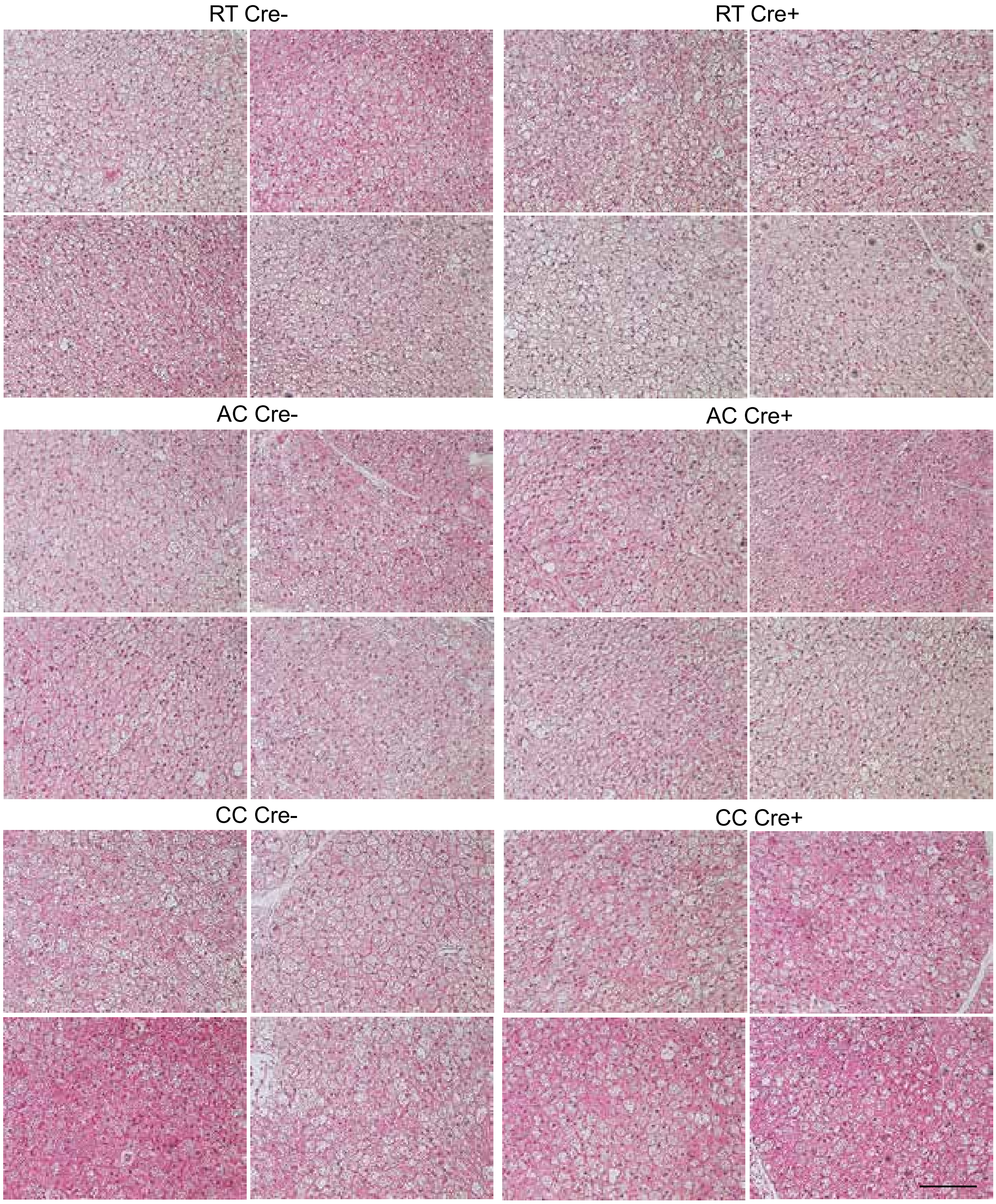

Figure S1A-B: BAT tissue weight and appearance. **A** iBAT weights (one-way Anova, different letters indicate p value below 0.05) and **B** Haematoxylin & eosin staining of BAT from Cre- and Cre+ mice housed at RT, AC or CC. Representative picture per mouse of n=4 pictures are shown. Bar corresponds to 100 µm.

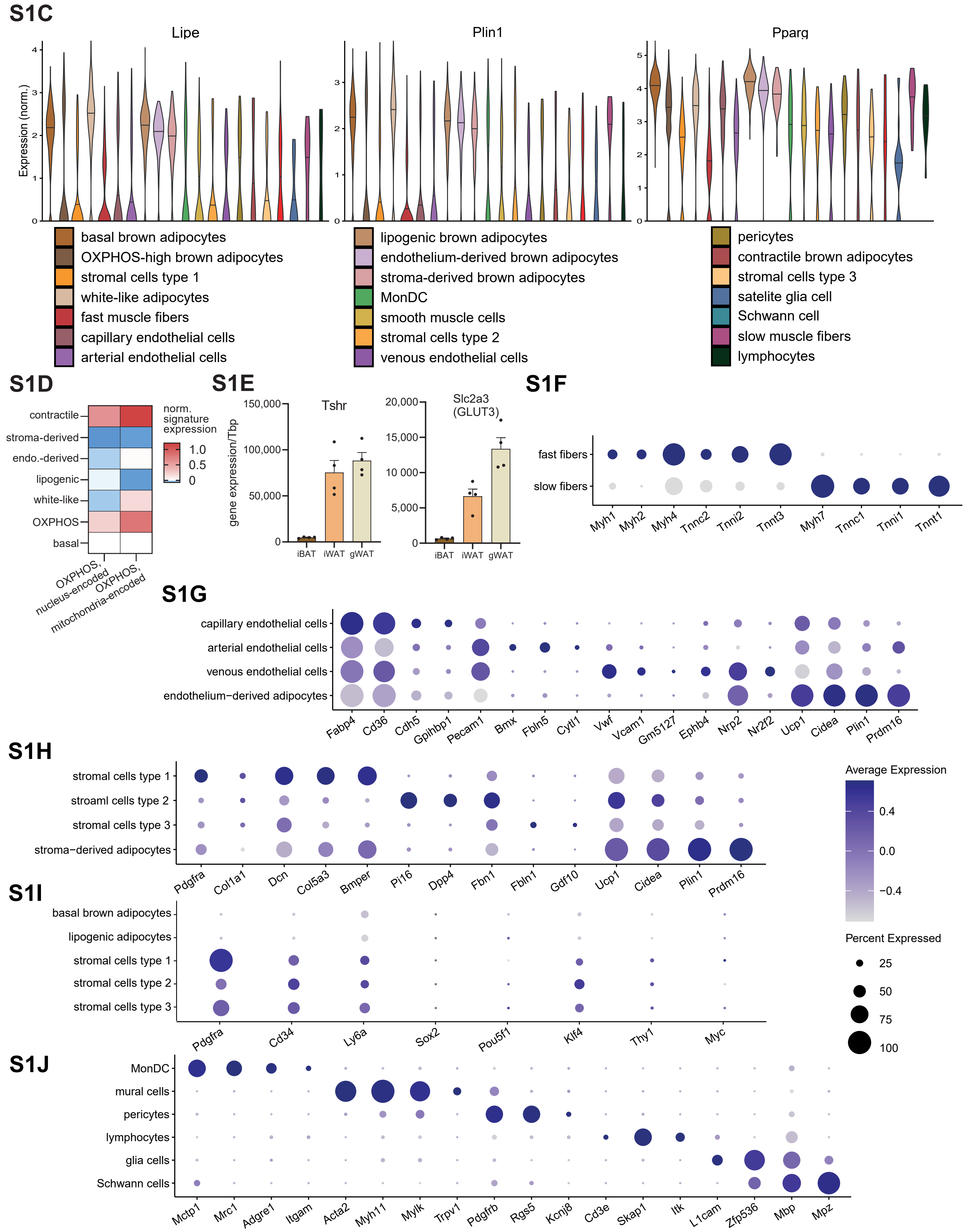

Figure S1C-J: Identification of cell types based on gene expression. Unless indicated otherwise, snRNA-seq data were used for the analysis shown in the graphs. **C** Normalized expression of adipocyte marker genes, determined in Cre- mice housed at RT. **D** Normalized signature expression of oxidative phosphorylation (OXPHOS) genes in the adipocyte subtypes. Data from Cre- mice housed at RT. **E** mRNA expression of Tshr and Slc2a3 measured by qPCR in iBAT, iWAT (inguinal) and gWAT (gonadal) of Cre- mice housed at RT. Mean±SEM. **F-H, J** Marker gene average expression and percentage of expression in muscle fibers (**F**), endothelial cells and endothelium-derived adipocytes (**G**), stromal cells and stroma-derived adipocytes (**H**) and other cell types (**J**) of Cre- mice at RT. **I** Stemness marker average expression and percentage of expression.

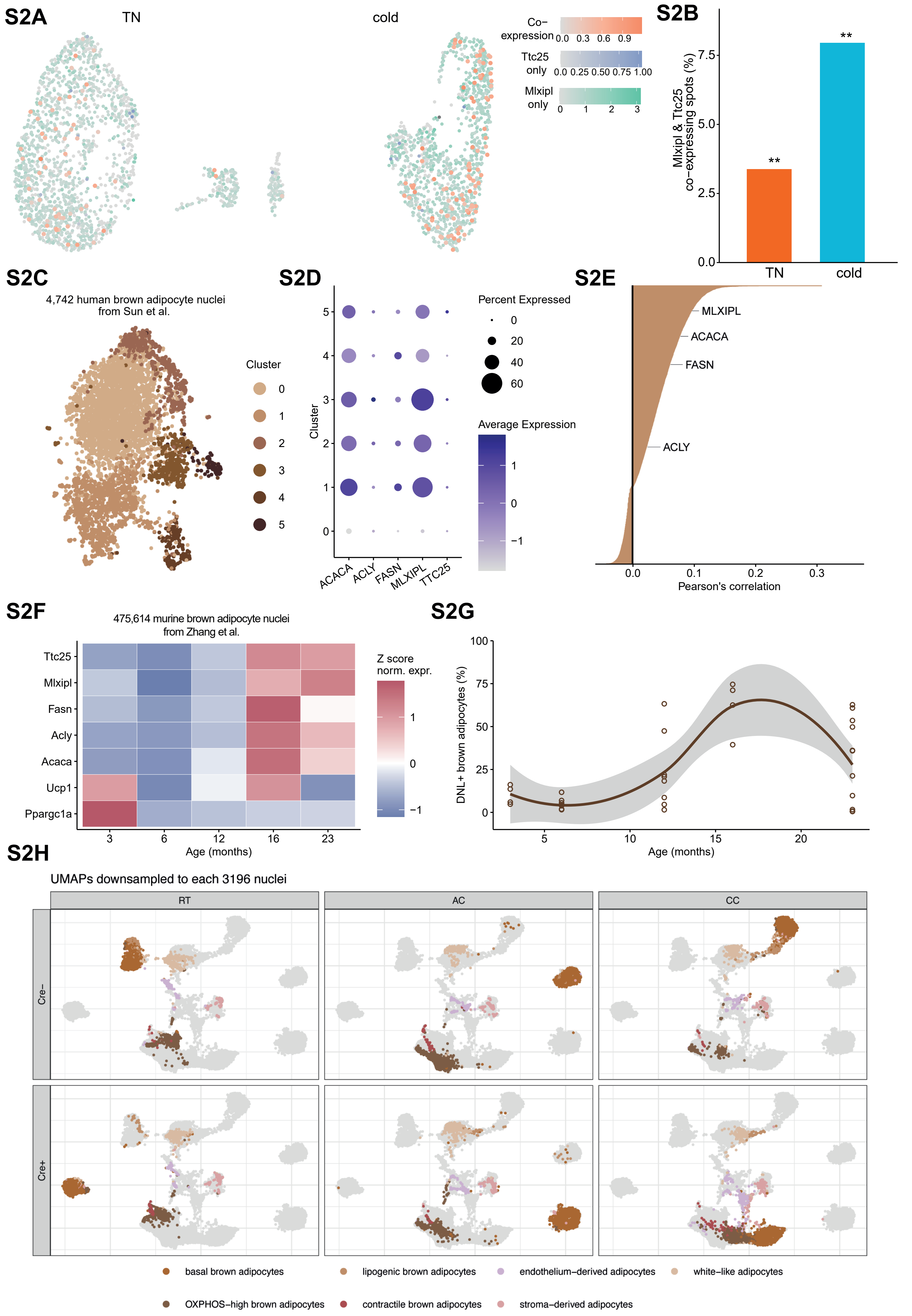
S2A
TN
cold
Co−expression
Ttc25 only
Mlxipl only
S2B
Mlxipl & Ttc25 co−expressing spots (%)
TN
cold
S2C
4,742 human brown adipocyte nuclei from Sun et al.
Cluster
S2D
Percent Expressed
Average Expression
ACACA
ACLY
FASN
MLXIPL
TTC25
S2E
MLXIPL
ACACA
FASN
ACLY
Pearson's correlation
S2F
475,614 murine brown adipocyte nuclei from Zhang et al.
Ttc25
Mlxipl
Fasn
Acly
Acaca
Ucp1
Ppargc1a
Z score norm. expr.
Age (months)
S2G
DNL+ brown adipocytes (%)
Age (months)
S2H
UMAPs downsampled to each 3196 nuclei
RT
AC
CC
Cre−
Cre+
basal brown adipocytes
lipogenic brown adipocytes
endothelium−derived adipocytes
white−like adipocytes
OXPHOS−high brown adipocytes
contractile brown adipocytes
stroma−derived adipocytes

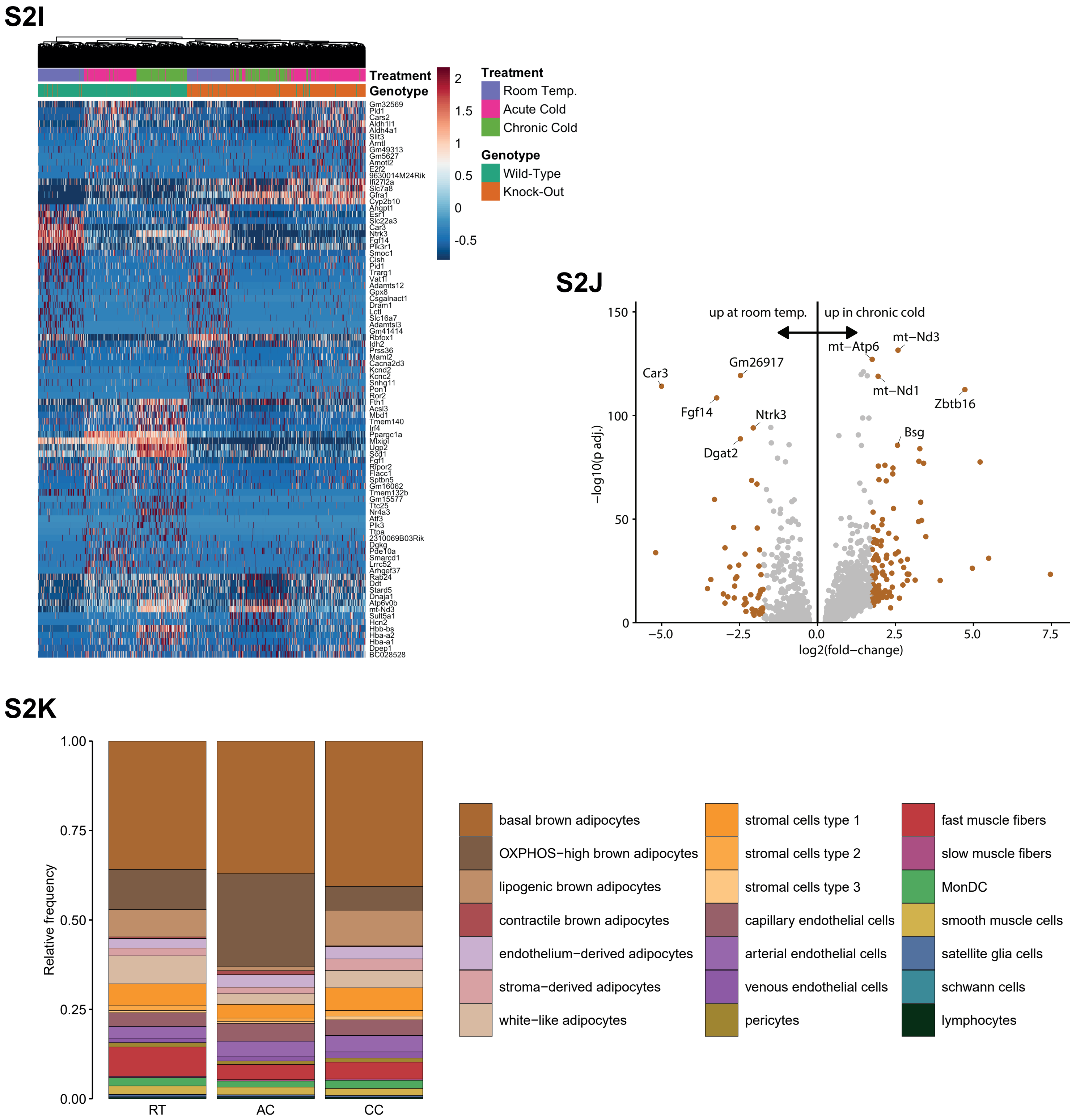

Figure S2: Relevance of lipogenic brown adipocytes. A-B Spatial co-expression analysis of Mlxipl and Ttc25 in murine BAT. Spatial transcriptomics data from Lundgren et al. [28] analyzed for co-expression patterns. **A** Percentage of spots showing co-expression of Mlxipl and Ttc25 in BAT from mice at thermoneutrality (TN) or after one cold-reactivation cycle (1cyc). Spots were considered positive for expression when normalized counts > 0. Permutation test (10.000 permutations) **p < 0.01, ***p < 0.001. Enrichment values indicate fold-change of observed co-expression over expected by chance. **B** UMAP of data in A. Spots are colored by expression intensity of Mlxipl only (green gradient), Ttc25 only (purple gradient), or co-expression of both genes (orange gradient). Gray indicates cells with neither gene expressed. **C** UMAP of 4,742 human brown adipocyte nuclei from Sun *et al*. (E-MTAB-8564). **D** Bubble plot of selected genes across the six human BAT adipocyte clusters. **E** Waterfall plot of Pearson correlation of genes from cluster 1 & 3 (from D) with TTC25. **F** Heatmap of scaled median expression of selected genes across 475,614 murine brown adipocyte nuclei during murine aging from Zhang *et al.* (GSE247719). **G** Age-dependent change of proportion of de novo lipogenesis (DNL)-positive brown adipocytes (from D). Cells with DNL scores above the 75th percentile were classified as DNL-positive. Each dot represents one mouse, loess-smoothed spline indicates the trend over time. **H** UMAP of subclustered adipocytes from snRNA-seq data of this study, splity by condition and downsampled to each 3,196 nuclei. Grey shadows indicate all nuclei. **I** Heatmap showing scaled expression (row-wise Z-scores) of the top 15 marker genes per condition in basal brown adipocytes, selected based on highest average $\log_2$ fold-change. Columns represent individual cells, annotated by genotype and treatment. Gene expression was capped at the 5th and 95th percentiles for visualization. Row and column hierarchies were clustered using Euclidean distance. **J** Volcano plot from snRNA-seq data of this study showing differentially expressed genes in lipogenic brown adipocytes from Cre- mice comparing chronic cold to room temperature. Genes with adjusted p-value < 0.05 and absolute of log2 fold-change > 1.96 × standard deviation are highlighted in brown. Each top five genes are labeled. **K** Relative frequencies of clusters across housing conditions of Cre- mice.

S3

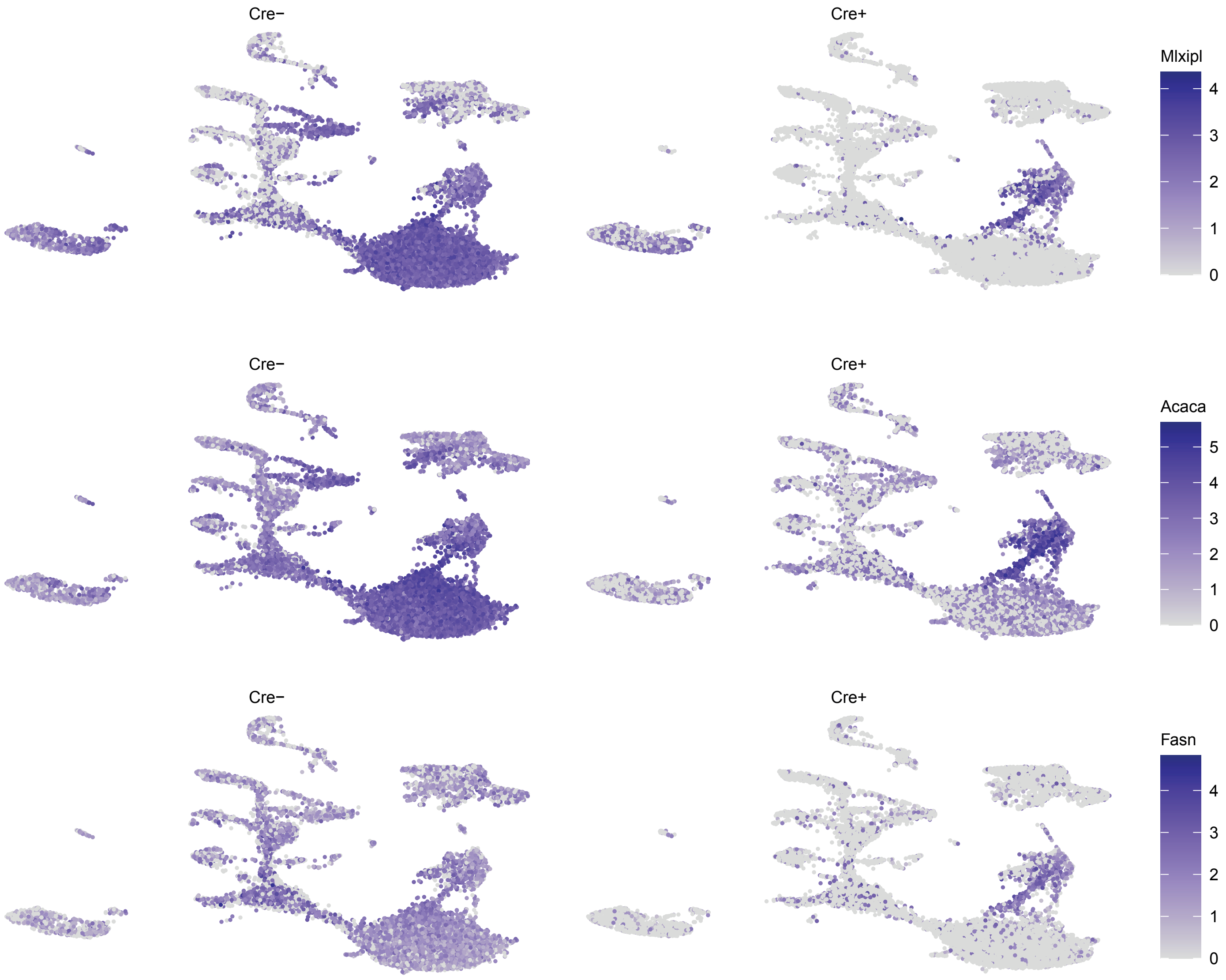

Figure S3. DNL gene expression in presence and absence of ChREBP. Normalized snRNA-seq data from all conditions (RT, AC, CC) were combined to compare Cre- with Cre+ mice.

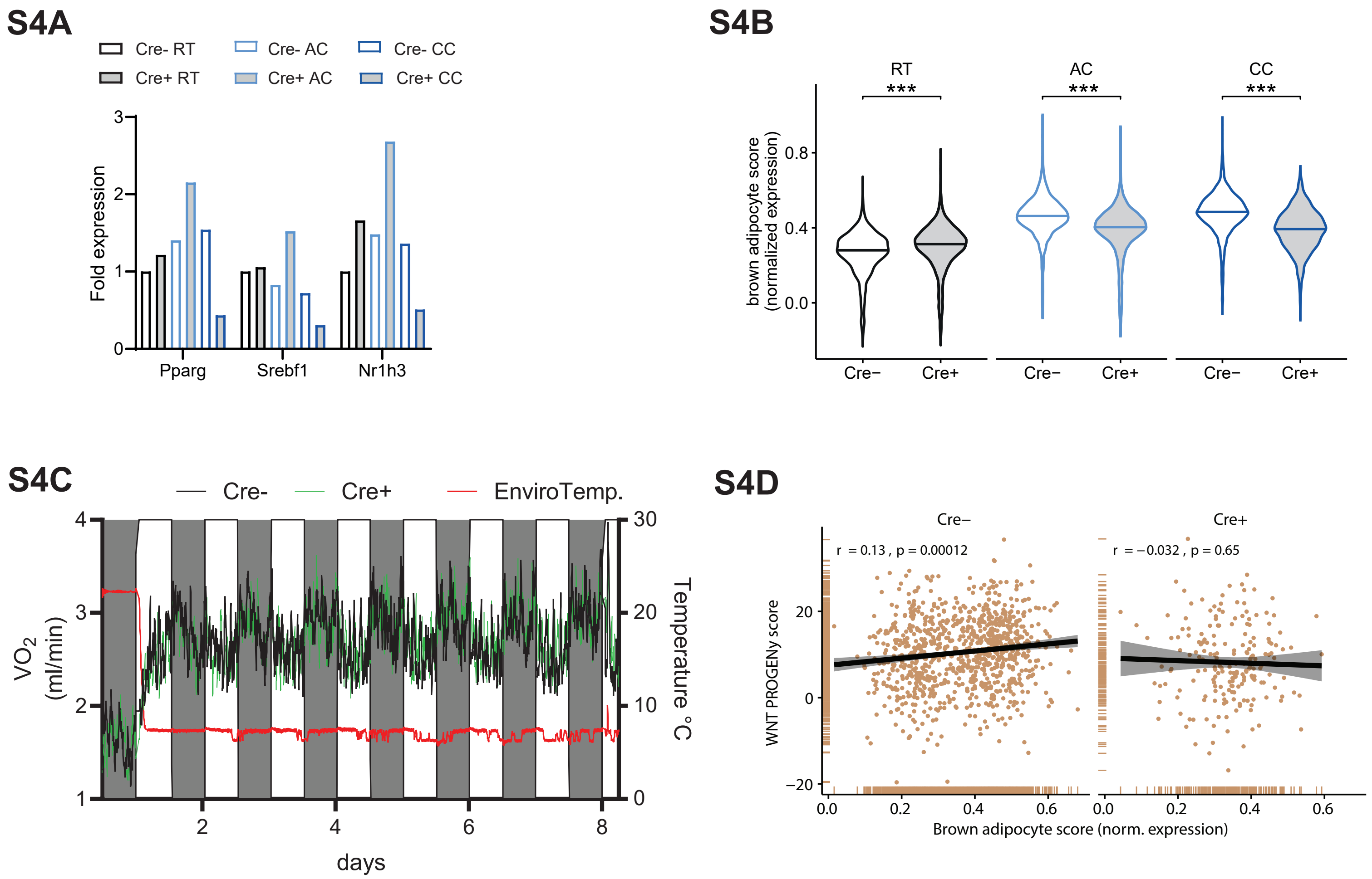

Figure S4. Thermogenic gene expression and energy expenditure in presence and absence of ChREBP. A Fold change of aggregated expression of *Pparg*, *Srebf1* and *Nr1h3* determined by snRNA-seq. **B** Violin plots of brown adipocyte score (based on Perdikari et al. [85]) Wilcoxon test, fdr-correction, *p < 0.05, **p < 0.01, ***p < 0.001. **C** Oxygen consumption rate (VO2 in ml/min) measured by indirect calorimetry of Cre- and Cre+ mice. **D** Pearson correlation between WNT PROGENy score and brown adipocyte score in lipogenic adipocytes of Cre- and Cre+ mice. Nuclei of the three housing conditions were combined. snRNA-seq data were used for **B** and **D**.
